# Duodenal and pancreatic duct strictures after severe acute pancreatitis successfully treated using fully covered duodenal metal stent-assisted ultrathin gastroscopy and duodenoscopy

**DOI:** 10.1055/a-2934-8416

**Published:** 2026-08-21

**Authors:** Jiawei Zhou, Juan Wei, Yuping Qiu, Changqing Sun, Huabing Xu, Ji Xuan

**Affiliations:** 1 Department of Gastroenterology, Jinling Hospital, Affiliated Hospital of Medical School, Nanjing University School of Medicine 12579 Southeast UniversityNanjingJiangsuChina; 2 Department of Gastroenterology 144990 Jinling Hospital, Affiliated Hospital of Medical School, Nanjing UniversityNanjingJiangsuChina; 3 Department of Gastroenterology 12461 Jinling Hospital, Nanjing Medical UniversityNanjingChina

Fully covered self-expandable metal stents (FCSEMSs) are widely used for refractory benign gastrointestinal strictures because they are retrievable and minimally invasive. 1​

**Fig. 1:**
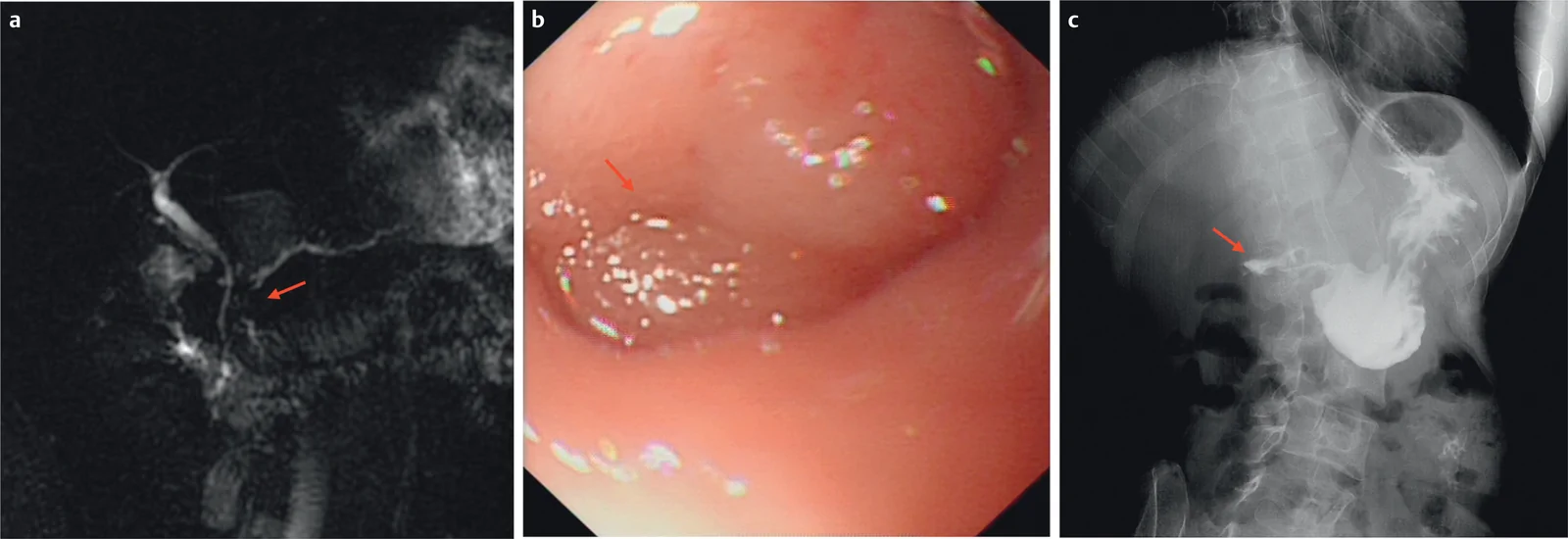
( **a** ) MRCP revealed a pancreatic duct stricture. ( **b** ) Endoscopy revealed a duodenal stricture. ( **c** ) Upper gastrointestinal radiography showed the delayed passage of the contrast medium through the duodenal region.

A 37-year-old patient developed duodenal and pancreatic duct strictures secondary to severe acute pancreatitis. Despite conservative therapy, resumption of oral intake repeatedly triggered recurrent pancreatitis with elevated amylase levels, abdominal pain, distension, and vomiting. Magnetic resonance cholangiopancreatography (MRCP) revealed a pancreatic duct stenosis ( Fig. 1a ). Endoscopy and upper gastrointestinal radiography confirmed a duodenal stricture ( Fig. 1b, c ).

**Fig. 2:**
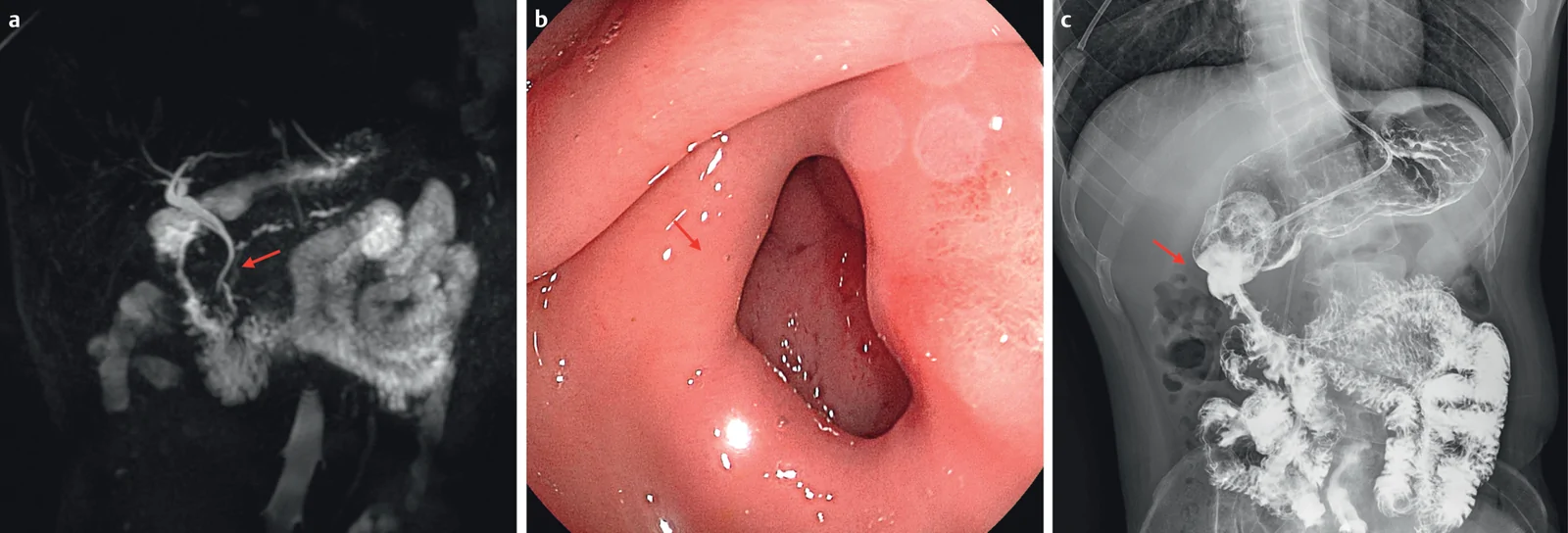
( **a** ) MRCP revealed an improvement in distal pancreatic duct dilatation with the restored visualization of the pancreatic duct at the neck after the treatment. ( **b** ) Endoscopy revealed the improvement of the duodenal stricture. ( **c** ) Upper gastrointestinal radiography showed patency after the treatment.

Surgery was not indicated. Endoscopic retrograde cholangiopancreatography (ERCP) was considered an option for recurrent acute pancreatitis with imaging-evident pancreatic duct outflow obstruction. 2​ As the duodenal stricture precluded conventional ERCP, a staged strategy was adopted in which a FCSEMS was deployed as a temporary bridge to secure luminal patency for subsequent pancreatic duct intervention, rather than as definitive therapy. Two weeks after resolution of the latest pancreatitis episode, a FCSEMS was placed under fluoroscopic guidance and anchored with four removable clips and a double-pigtail biliary stent. Multiple fenestrations were created in the membrane over the papilla using a papillotome and a guidewire to maintain biliopancreatic drainage. Postprocedural bilirubin and amylase levels remained normal. Two weeks later, the clips and pigtail stent were removed. A duodenoscope was advanced through the stent lumen to the papilla while an ultra-thin gastroscope simultaneously retracted the stent cephalad via its retrieval suture, thereby exposing the papilla. Pancreatography revealed a pancreatic head‑neck stricture, which was dilated with a 5‑Fr bougie and stented. The duodenal stent was then removed and a jejunal feeding tube was placed ( Video 1 ). One month later, the patient tolerated a normal diet without symptom recurrence, with improved MRCP, endoscopic, and radiographic findings ( Fig. 2a–c ).

**Video 1** Duodenoscopy combined with ultra-thin gastroscopy engaged the retrievable suture of the stent to expose the papilla for ERCP.


This staged FCSEMS-assisted dual‑endoscopy approach may provide a feasible strategy for patients with concomitant duodenal and pancreatic duct strictures when conventional ERCP is not feasible.

Endoscopy_UCTN_Code_TTT_1AR_2AI
